# A Review of Pancreatico-Pleural Fistula in Pancreatitis and Its Management

**DOI:** 10.1155/1992/90415

**Published:** 1992

**Authors:** N. A. Burgess, H. E. Moore, J. O. Williams, M. H. Lewis

**Affiliations:** East Glamorgan General Hospital Mid. Glam Pontypridd UK

## Abstract

Pancreatico-pleural fistula is a rare condition in which pancreatic enzymes drain directly in to the pleural cavity, most commonly from an enlarging pseudocyst. We review the literature on the causes, investigations and treatment of pancreatico-pleural fistulae and compare this with our own experience of the case of a 41 year old man with a left sided pancreatico-pleural fistula associated with pancreatic duct obstruction. The fistula could not be demonstrated by USS, CT or ERCP, and after these investigations the patient was managed conservatively. However, deterioration in the patients' condition led to an urgent but not emergency laparotomy and operative pancreatogram. This demonstrated the distally obstructed pancreatic duct, with associated pleural fistula for which aggressive surgical intervention was indicated. The patient subsequently completely recovered.


*HPB Surgery*, 1992, Vol. 5, pp. 79–86
Reprints available directly from the publisher
Photocopying permitted by license only

© Harwood Academic Publishers GmbH
Printed in the United Kingdom

### REVIEW ARTICLE

### A REVIEW OF PANCREATICO-PLEURAL FISTULA IN PANCREATITIS AND ITS MANAGEMENT

N. A. BURGESS, H. E. MOORE, J. O. WILLIAMS and M.H. LEWIS
*East Glamorgan General Hospital, Pontypridd, Mid. Glam*

*(Received 1 September 1991)*

Pancreatico-pleural fistula is a rare condition in which pancreatic enzymes drain directly in to the pleural cavity, most commonly from an enlarging pseudocyst.

We review the literature on the causes, investigations and treatment of pancreatico-pleural fistulae and compare this with our own experience of the case of a 41 year old man with a left sided pancreatico-pleural fistula associated with pancreatic duct obstruction.

The fistula could not be demonstrated by USS, CT or ERCP, and after these investigations the patient was managed conservatively. However, deterioration in the patients' condition led to an urgent but not emergency laparotomy and operative pancreatogram. This demonstrated the distally obstructed pancreatic duct, with associated pleural fistula for which aggressive surgical intervention was indicated. The patient subsequently completely recovered.

KEY WORDS: Pancreatitis, pancreatico-pleural fistula

### CASE HISTORY

A 41 year old ex-colliery worker with a past history of alcohol abuse and pancreatitis was admitted as an emergency to a general medical firm. He had a two week history of shortness of breath, especially in the mornings, one week of a cough productive of white sputum and four days of left sided pleuritic chest pain radiating to his left shoulder.

He had been previously diagnosed as having gall stones and several years earlier had a documented pseudocyst noted on a computed tomography scan. This cyst had settled with conservative management.

On examination he was pyrexial, dyspnoeic and tachypnoeic, with dullness and decreased air entry at the base of his left lung. He also had a tense tender abdomen. Blood tests showed normal urea, electrolytes, leucocyte count and clotting screen.

Although he denied excess alcohol intake for the 18 months prior to admission, he had a macrocytic anaemia with a haemoglobin of 10.8g/dL, mean cell volume of 102.6 and a reticulocyte count of 3%. Amylase was 256 IU/L (normal range = 0 - 90 IU/L), bilirubin was normal with a mildly raised alkaline phosphatase 183 IU/L (normal range = 30 - 120 IU/L) and raised alanine transaminase 461 IU/L (normal range = 0 - 50 IU/L). His erythrocyte sedimentation ratio was 92.

---

Address correspondence to: Mr. M.H. Lewis, East Glamorgan General Hospital, Pontypridd, Mid. Glam

A chest X-ray revealed a large left pleural effusion (Figure 1) from which initially two litres of fluid were drained by aspiration. Analysis of the aspirate revealed an amylase of 15,320 IU/L. An abdominal ultrasound scan showed multiple small gall stones but no evidence of pancreatic pseudocyst or pancreatic duct dilatation. Over the next 10 days he developed a persistent low grade pyrexia and increased left shoulder tip pain.

His serum amylase increased to 570 IU/L and a further litre of pleural fluid was aspirated. At 12 days following his admission he was referred for a surgical opinion, when a further three litres of pleural fluid were drained. Pleural amylase content was 37,000 IU/L with a serum amylase of 636 IU/L.

A CT scan showed a persistent large, left pleural fluid collection causing compressive collapse of most of the left lung, displacement of the mediastinum to the right and the diaphragm inferiorly, in addition an abdominal fluid collection, remote from the tail of the pancreas, was displacing the spleen anteriorly and inferiorly, however, this was not confirmed at laparotomy. The pancreatic duct was not seen but there was calcification in the tail of the pancreas, and calcified gall stones in the gall bladder.

At this time the ultrasound scan revealed a 13mm cyst in the tail of the pancreas and gall stones (Figure 2).

Within two days of his transfer to the surgical ward (i.e. 15 days after initial admission) he developed endotoxic shock with increasing dyspnoea, haemoglobin of 12.2 g/dL, leucocyte count of 31.5 × 10/L and arterial blood gases showed a metabolic acidosis. His serum amylase had also increased to 1252 IU/L. He was transferred to the intensive care unit and a chest drain inserted into the left hemithorax, to allow free and continuous drainage. He was commenced on antibiotics, oxygen and a haemacell infusion. During the next 24 hours his haemoglobin dropped to 9.6 g/dL and he was transfused 6 units of blood.

Endoscopic retrograde chloangio pancreatography was undertaken which showed a normal proximal duct but a slightly dilated and truncated duct in the tail of the pancreas (Figure 3). There was some extravasation of contrast through a cystic structure lying superior to the duct in the tail, but there was no evidence of contrast passing to the left hemithorax.

He was prepared for urgent, but not emergency surgery within five days of his surgical referral. He underwent laparotomy where gall stones were identified in the gall bladder. The head of the pancreas appeared normal but the tail was distended. An on-table pancreatogram was performed via a direct puncture of the mid pancreatic duct using a butterfly needle (23FG) and 15ml of contrast material. This demonstrated a normal proximal duct and drainage mainly via the accessory duct to the duodenum, but distally the duct was dilated and contained stones (Figure 4). There were no stones identified in the common bile duct. There was a fistula passing through the perirenal fat, superiorly into the left chest.

A cholecystectomy, distal pancreatectomy and closure of the fistula was performed, the spleen was found to be partly necrotic and adherent and was therefore also removed. He made an uneventful recovery and was discharged home with strong advice to continue to avoid alcohol.

Histology revealed changes of chronic pancreatitis with no neoplastic change, and mild cholecystitis.

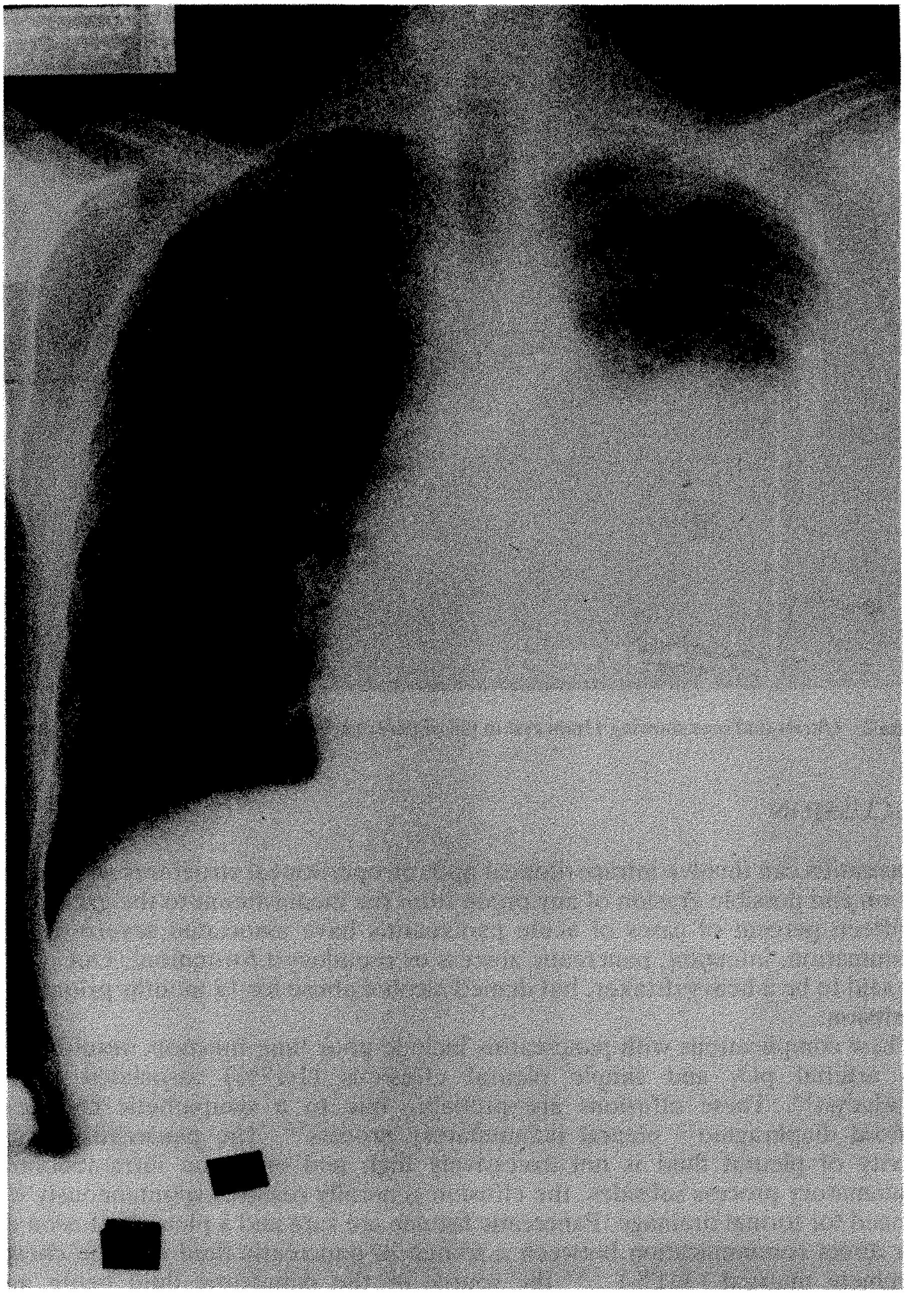

**Figure 1** Chest X-ray showing large left pleural effusion.

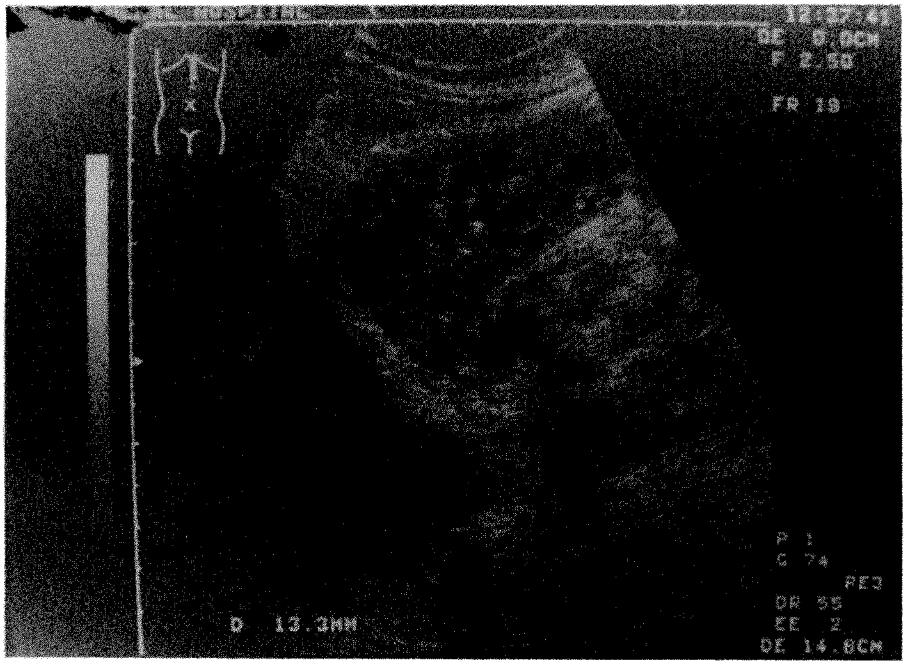

**Figure 2** Ultrasound scan showing 13mm cyst in tail of pancreas lying in close proximity to diaphragm.

### DISCUSSION

Pancreatitis can involve intraperitoneal and retroperitoneal structures with inflammation and possible erosion of any organ from the mediastinum to the groin[1]. Five to fifteen percent of cases of acute pancreatitis have associated severe necrotic inflammation, infection, pancreatic abscess or pseudocyst formation[2]. Our patient was said to be a heavy drinker, but denied alcohol abuse for 18 months prior to this admission.

Chest complications with pancreatitis include poor lung function, resulting in a low arterial $pO_2$ and simple pleural effusions (1-17%) associated with a pseudocyst[3,4]. These effusions are probably due to a sympathetic chemically induced diaphragmo - pleural inflammatory process[5,6]. The pancreatic enzyme activity of pleural fluid is not excessively high and once the intra-abdominal inflammatory process subsides, the effusion generally resolves spontaneously with no need for formal drainage. Pancreatic fistulae are rare and a pleural effusion due to a direct communication between a source of pancreatic fluid and the chest is extremely unusual (<1%) [7,8,9] the source of the pancreatic fluid being most commonly, a pseudocyst.

Anterior duct disruption can result in pseudocyst formation, as escaping fluid is walled off by surrounding viscera in the lesser sac[2,10]. A posterior leak collects retroperitoneally and has the potential to seal off or fistulate into the chest[11,12,13,9].

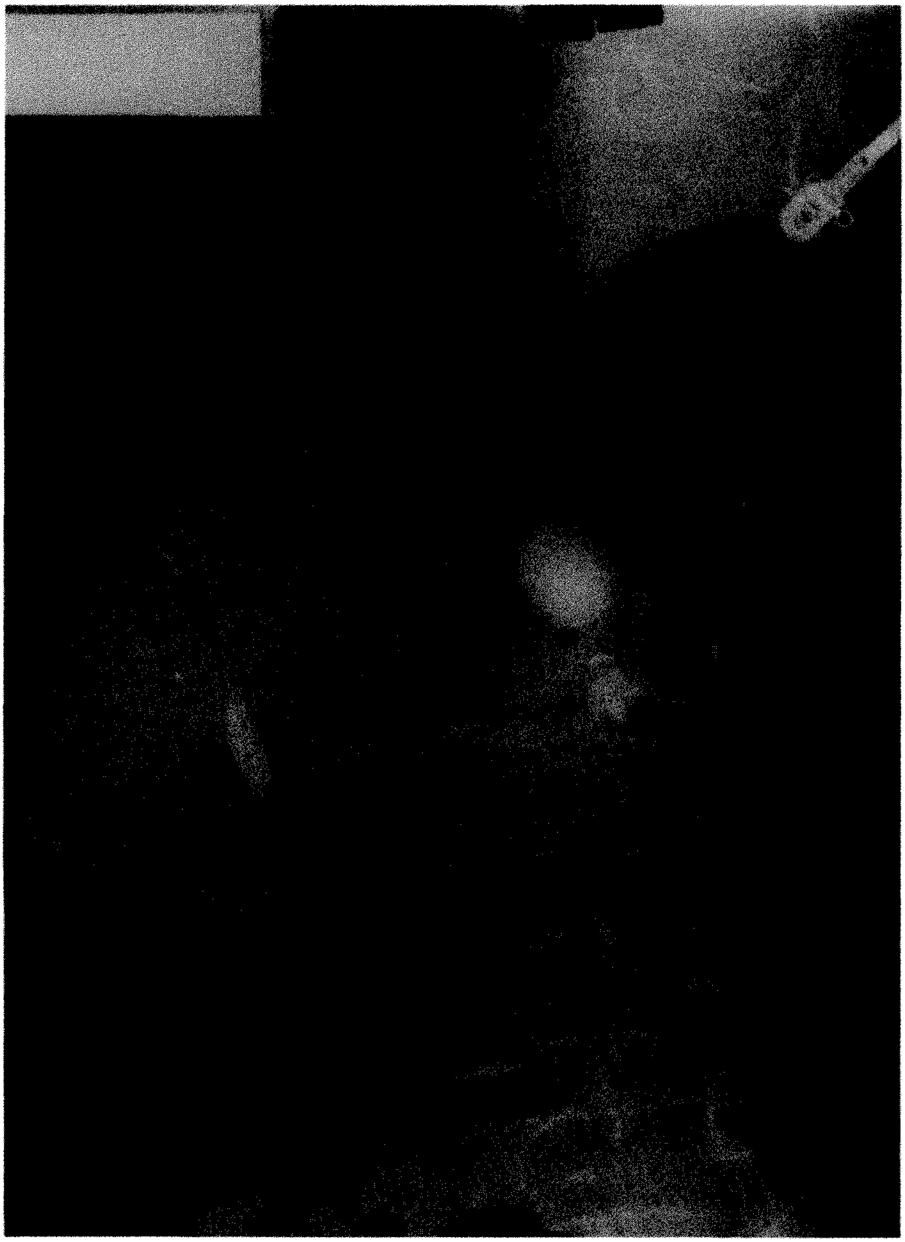

**Figure 3** Pre-op ERCP showing normal proximal pancreatic duct but a slightly dilated duct in the tail of the pancreas with extravasation of contrast through a cystic structure lying superior to the pancreatic duct.

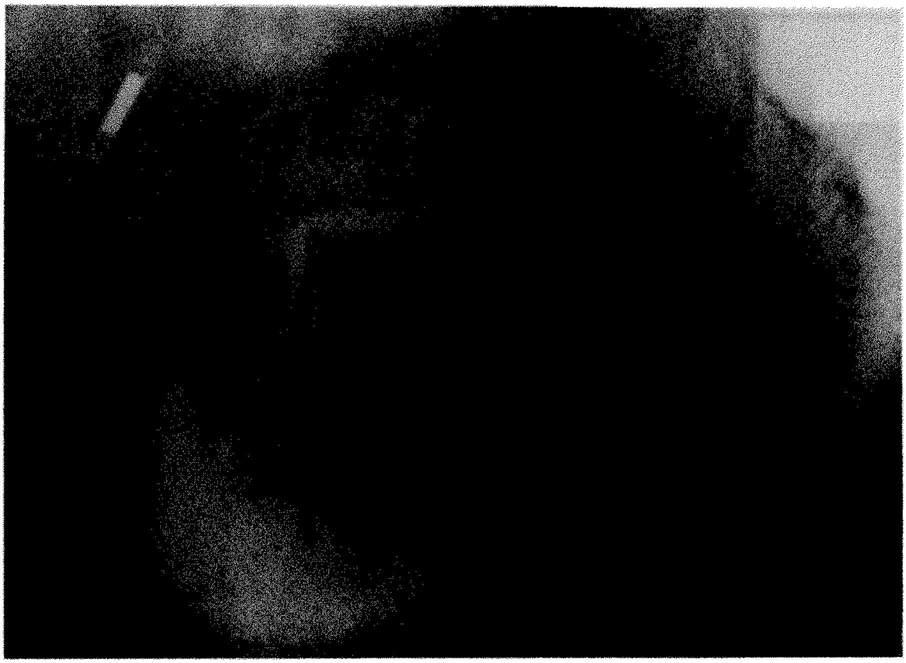

**Figure 4** Operative panceratogram demonstrating a normal proximal duct and drainage mainly via the accessory duct into the duodenum.

Persistent communication between pancreatic duct system and the pseudocyst, can cause it to enlarge and spread along fascial planes of least resistance, for example, along the leaves of the transverse mesocolon or greater omentum[7].

These collections can be very large, the pancreas producing over a litre of fluid per day. Psudocyst rupture, or anterior duct disruption without visceral walling off can result in pancreatic ascites[10,9]. A pancreatic pleural effusion develops due to a direct passage through a natural hiatus in the diaphragm into the mediastinum (e.g. oesophageal or aortic hiatus) or by direct penetration through the dome of the diaphragm ignoring normal fascial planes[6].

In our patient, although there was no sign of a recent pseudocyst, it is interesting to speculate that he may have developed the fistula from spontaneous rupture of his previously documented cyst, although this seems unlikely because he had been well for the three years following discovery of the cyst with normal ultrasound scans and chest X-ray. At operation, the fistulous tract was found passing directly from an obstructed pancreatic duct, over the anterior surface of the left kidney and beneath the arcuate ligament of the left hemidiaphragm. A pleural fistula from the pancreas is often difficult to diagnose, the main physical signs being of an effusion — cyanosis, dullness to percussion and decreased breath sounds, all signs easily confused with pulmonary embolus or infection.

Abdominal symptoms are often few[11,14,2,15,16]. Therefore, in cases of unexplained pleural effusion especially with a past history of alcohol abuse or chronic pancreatitis, pleural aspirate pancreatic enzyme activity should be measured, high levels being diagnostic of an internal pancreatico-pleural fistula[3,13,6], as it was in our patient. Positive absorption of amylase from pleural fluid to serum results in an elevated serum amylase at a level lower than the pleural fluid amylase[12,4]. In our patient the serum amylase was 570 IU/L and the pleural 37,000 IU/L.

Subsequent radiological intervention by ERCP is required to demonstrate the ductal segment[6,12,13,16].

Visualisation of the entire ductal tree is essential in planning a rational surgical approach, however, ERCP may often provide inadequate information. In our patient it was of some value, in that it demonstrated disruption of the main pancreatic duct and extravasation of contrast material through a cystic structure in the tail of the pancreas above the distal duct, but it did not demonstrate the dilated distal duct itself which also contained pancreatic stones. One possible reason why the fistula could not be demonstrated by ERCP may be that there was an obstruction in the main pancreatic duct in the tail of the gland and this has been previously reported[17]. Computerised tomography and sonography are also recommended, but in our case, apart from some evidence of chronic pancreatitis, they did not help to determine the origin of the fistulous tract, this was only demonstrated by an operative pancreatogram. This investigation showed complete obstruction to the mid pancreatic duct with dilatation and pancreatic stones in the distal pancreas. In addition, it delineated the point at which the main duct returned to normal calibre proximally, with some drainage to the duodenum by the accessory duct. Operative pancreatography is simple to perform, requiring no specialised apparatus and with care is a safe investigation[17]. Management of pancreatic fistulae is still controversial[18,2,13]. Many authors recommend an initial conservative approach progressing to a surgical approach if this fails. However, a pancreatico-pleural fistula associated with persistent duct obstruction preventing normal pancreatic fluid drainage cannot be expected to heal spontaneously.

Delaying treatment may lead therefore to severe sepsis as it did in our patient, or development of a pancreatico-bronchial or pancreatico-pleural fistula. We therefore suggest that a more aggressive approach to diagnosis and treatment are indicated as did Izbicki (1980)[11]. He recommended conservative treatment only if an ERCP did not reveal any ductal pathology. However, no ductal pathology was revealed following ERCP in our patient, because of underfilling secondary to stenosis of the pancreatic duct in the mid body of the gland. This led to a delay in operation and a significant deterioration in his condition. We therefore, cannot agree with Izbicki's policy.

Fistulae from a pseudocyst which is no longer in direct communication with the pancreatic ductal system, may heal spontaneously, but ERCP, CT scan and USS are not always sufficient in providing accurate radiological delineation of the system and laparotomy and an operative pancreatogram plays a major role in determining the operative procedure. Ductal visualisation is crucial in deciding between a resection or a drainage procedure and provides an accurate depiction of associated cysts or fistulae[17].

If there is obstruction of a main pancreatic duct proximal to the fistula, surgical treatment is mandatory to decompress the stenotic or obstructed duct, with or without excision of the involved portion of the obstructed pancreas[9]. A proximal stenosis, with distally dilated ducts requires a pancreatico-jejunostomy. A distal duct obstruction, as in our patient, can be treated by pancreatic resection proximal to the obstruction — determined accurately by an operative pancreatogram (Fielding, 1989)[2]. These procedures are associated with good long and short term results[19,10]. Repair of the fistulous tract, where it extends into the chest is unnecessary, as removal of the obstruction and disruption of the pancreatic duct will prevent further passage of fluid into the chest.

In summary, prompt recognition of a pancreatico-pleural fistula is very important, as delay in surgical treatment is associated with a high morbidity and mortality. Therefore, pancreatic enzyme assays of pleural effusion of obscure cause should be considered early and if elevated levels are detected, active radiological investigations with CT, USS and ERCP should precede timely surgical intervention. However, an operative pancreatogram may provide the only truly accurate picture and if ductal obstruction is present due to ductal stenosis, release of the obstruction is essential, allowing the fistula to heal with a good long term prognosis.

### *References*

1. Martin, F.M., Rossi, R.C., Munson, J.L., Remine, S.G. and Braasch, J.W. (1989) Management of pancreatic fistulas. *Arch. Surg.*, **1241**, 571–573
2. Fielding, G.A., McLatchie, G.R., Wilson, C., Imrie, C.W. and Carter, D.C. (1989) Acute pancreatitis and pancreatic fistula formation. *Br. J. Surg.*, **76**, 1126–1128
3. Frey, C.F. (1978) Pancreatic pseudocyst - operative strategy. *Ann. Surg.*, **188**(5), 652–662
4. Miridjanian, A., Ambrvoso, V.N., Derby, B.M. and Tice, D.A. (1969) Massive bilateral haemorrhagic pleural effusion in chronic relapsing pancreatitis. *Arch. Surg.*, **98**, 62–67
5. Tombroff, M., Lok, A., De Koster, J.P., Engleholm, L. and Govaerts, J.P. (1973) Pleural effusion with pancreatico-pleural fistula. *Br. Med. J.*, **1**, 330–331
6. Kaye, M.D. (1968) Pleuropulmonary complications of pancreatitis. *Thorax*, **23**, 297–306
7. Stone, M.M., Stone, N.N., Meller, S. and Kim, U. (1989) Bilateral ureteric obstruction an unusual complication of pancreatitis. *Am. J. Gastroenterology*, **84**(1), 49–51
8. Grossman, A., Jackson, B.T., Thompson, R.P.H. and Brainbridge, M.V. (1978) Pancreatico-broncho-pleural fistula as a complication of acute pancreatitis. *Br. J. Clin. Prac.*, **32**, 298–301
9. Portmeyer III, E.W., Frey, C.F. and Matsuno, S. (1987) Pancreaticopleural fistulas. *Arch. Surg.*, **122**, 648–655
10. Cameron, J.L., Kieffer, R.S., Anderson, W.J. & Zuidema, G.D. (1976) Internal Pancreatic fistulas: pancreatic ascites and pleural effusion. *Ann. Surg.*, **184**(5), 587–593
11. Izbicki, J.R., Wilker, D.K., Waldner, H., Rueff, F.L. and Schweiberer, L. (1989) Thoracic manifestations of internal pancreatic fistulas: report of five cases. *Am. J. Gastroenterology*, **84**(3), 265–271
12. Marshall, J.P. (1982) Pancreatic ascites and pleural effusion. *Nebraska Med. J.*, **Sept**, 252–255
13. Davidson, E.D., Horney, J.T. and Salter, P.P. (1979) Internal pancreatic fistula to the pericardium and pleura. *Surgery*, **85**(4), 478–480
14. Kivilvoto, T., Kivisaari, L., Kivilaakso, E. and Lempinen, M. (1989) Pseudocysts in chronic pancreatitis — surgical results in 102 consecutive patients. *Arch. Surg.*, **124**, 240–243
15. Tewari, S.C., Jayaswal, R., Chauhan, M.S., Kaul, S.K. and Naryanan, V.A. (1989) Bilateral recurrent haemorrhagic pleural effusion in asymptomatic chronic pancreatitis. *Thorax* **44**, 824–825
16. Greenward, R.A., Deluca, R.f. and Raskin, J.B. (1979) Pancreatic pleural fistula. Demonstration by ERCP and successful treatment with radiation therapy. *Dig. Dis. Sci.*, **24**(3), 240–244
17. Cooper, M.J. and Williamson, R.C.N. (1983) The value of operative cholangiography. *Br.J.Surg.*, **70**, 577–580
18. McLatchie, G.R., Meek and Imrie, C.W. (1985) The use of ERCP in the diagnosis of internal fistulae complicating severe acute pancreatitis. *Br. J. Radiol.*, **58**, 395–397

*(Accepted by S. Bengmark 3 October 1991)*

